# Overview of research progress and application of experimental models of colorectal cancer

**DOI:** 10.3389/fphar.2023.1193213

**Published:** 2023-07-04

**Authors:** Li Liu, Qiuying Yan, Zihan Chen, Xiaoman Wei, Lin Li, Dongxin Tang, Jiani Tan, Changliang Xu, Chengtao Yu, Yueyang Lai, Minmin Fan, Lihuiping Tao, Weixing Shen, Liu Li, Mianhua Wu, Haibo Cheng, Dongdong Sun

**Affiliations:** ^1^ School of Integrated Chinese and Western Medicine, Nanjing University of Chinese Medicine, Nanjing, China; ^2^ Collaborative Innovation Center of Jiangsu Province of Cancer Prevention and Treatment of Chinese Medicine, Nanjing University of Chinese Medicine, Nanjing, China; ^3^ Research Center for Pathogenesis Theory of Cancerous Toxin and Application, Nanjing University of Chinese Medicine, Nanjing, China; ^4^ The First Clinical Medical College, Guizhou University of Traditional Chinese Medicine, Guiyang, China

**Keywords:** colorectal cancer, cellular models, animal models, preclinical studies, drug development

## Abstract

Colorectal cancer (CRC) is the third most common malignancy in terms of global tumor incidence, and the rates of morbidity and mortality due to CRC are rising. Experimental models of CRC play a vital role in CRC research. Clinical studies aimed at investigating the evolution and mechanism underlying the formation of CRC are based on cellular and animal models with broad applications. The present review classifies the different experimental models used in CRC research, and describes the characteristics and limitations of these models by comparing the research models with the clinical symptoms. The review also discusses the future prospects of developing new experimental models of CRC.

## 1 Introduction

Colorectal cancer (CRC) is the most common malignancy worldwide, in terms of both morbidity and mortality (Sung et al., 2021). The understanding of the origin of CRC has increased dramatically over the past few decades. However, despite breakthroughs in diagnosis and treatment, CRC continues to be a major health concern worldwide. The morbidity and mortality due to CRC are on the rise owing to the overall low screening rates and changes in lifestyle, including poor diets, irregular lifestyles, smoking, and other factors (Minami et al., 2022). Strategies for the early screening and intervention of precancerous CRC lesions in developed countries have reduced the rates of incidence and mortality due to CRC (Zorzi and Urso, 2022). Similar to studies on other illnesses, research studies on CRC critically depend on experimental models with reliable and distinct characteristics. Although CRC tumors have heterogeneous characteristics, experimental models of CRC are established in such a manner that they represent the characteristics of CRC tumors. Selection of the appropriate model that reflects the tumor system is a crucial challenge in cancer screening. Therefore, experimental models of CRC have been extensively studied for determining the optimum model for studying the invasion, progression, and early detection of CRC. This review discusses the significance of CRC models as a platform for screening drugs and developing novel therapeutic approaches for CRC. The application of cellular and animal models of CRC were also summarized and discussed to aid further preclinical studies on CRC.

## 2 Cellular models based on intestinal cells and CRC cells

*In vitro* models of CRC established using intestinal cells and CRC cells are frequently employed for obtaining rapidly growing cellular models of CRC and for facilitating experimental control. *In vitro* models of CRC can simultaneously generate several populations of homogeneous cells. Specific cellular targets of macroscopic systems can be conveniently studied using these models by analyzing the experimental results (Saeidnia et al., 2015).

**FIGURE 1:**
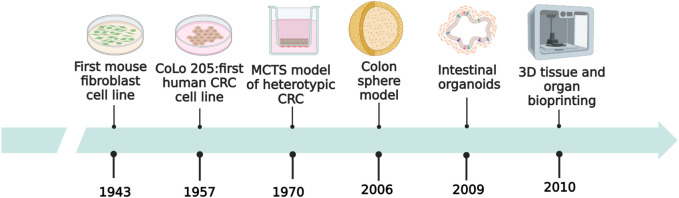
History of development of *in vitro* models of CRC.

The first mammalian cell line was established in 1943, which served as a prelude to *in vitro* cell culture. The CoLo 205 CRC cell line was established in 1957, which promoted *in vitro* studies on CRC. Figure 1 depicts the history of development of *in vitro* models of CRC (Sanford et al., 1948; Ricci et al., 2007; Sharma et al., 2010; Jedrzejczak, 2017).

### 2.1 Two-dimensional (2D) cellular models of CRC

CRC cell lines are *in vitro* tumor models with different origins and types, and serve as fundamental tools for investigating the biomarkers of drug sensitivity, resistance, and toxicity. CRC cell lines are established by isolating CRC cells from patients or animals with CRC followed by culture on artificial media. The appropriate cell lines are selected based on the type of cancer or gene expression levels, according to the aims of the study. SW620, Caco-2, RKO, SW480, HT8, HT29, HT116, LoVo, and LS174 T cell lines are currently widely used in basic research studies on CRC (Akashi et al., 2000; Vécsey et al., 2002; Lind et al., 2004; Barretina et al., 2012; Ahmed et al., 2013; Gemei et al., 2013; Mouradov et al., 2014; Maletzki et al., 2015; Boot et al., 2016; Berg et al., 2017; Mooi et al., 2018; Kim et al., 2020; Bian et al., 2021).

Although the characteristics of CRC cell lines are highly consistent with those of human cancer models, they have certain limitations. CRC cell lines facilitate the investigation of the molecular and phenotypic characteristics of CRC. However, as only one side of the cells is in contact with the medium during culture, the majority of cells gradually flatten, undergo abnormal division, and lose their differentiation phenotype following isolation from tissues and plate culture. Additionally, CRC cells continue to proliferate *in vitro*, which may cause the cell lines to lose the characteristics of the original tumor. Another limitation of CRC cell lines is the scarcity of matrix ingredients in the tumor microenvironment (TME), including the cells and acellular components constituting the structural complexity of the *in vivo* environment. Altogether, these indicate that CRC cell lines fail to accurately mimic the *in vivo* growth characteristics of tumor cells.

### 2.2 Three-dimensional (3D) cellular models of CRC

Owing to the limitations of 2D cellular models of CRC, researchers are committed towards exploiting novel and physiologically representative models of CRC. *In vitro* 3D culture models, including spheroids and organoids, are therefore used for overcoming the limitations of 2D cellular models. Spheroids comprise a mixture of single-cell or multicellular systems, while organoids are generally formed of specific stem cells or ancestral cells from organs (Kimlin et al., 2013; Boucherit et al., 2020). Spheroids and organoids are superior at mimicking tumor cell heterogeneity and the complex interactions among different cells (Thoma et al., 2014).

#### 2.2.1 Spheroids

**FIGURE 2:**
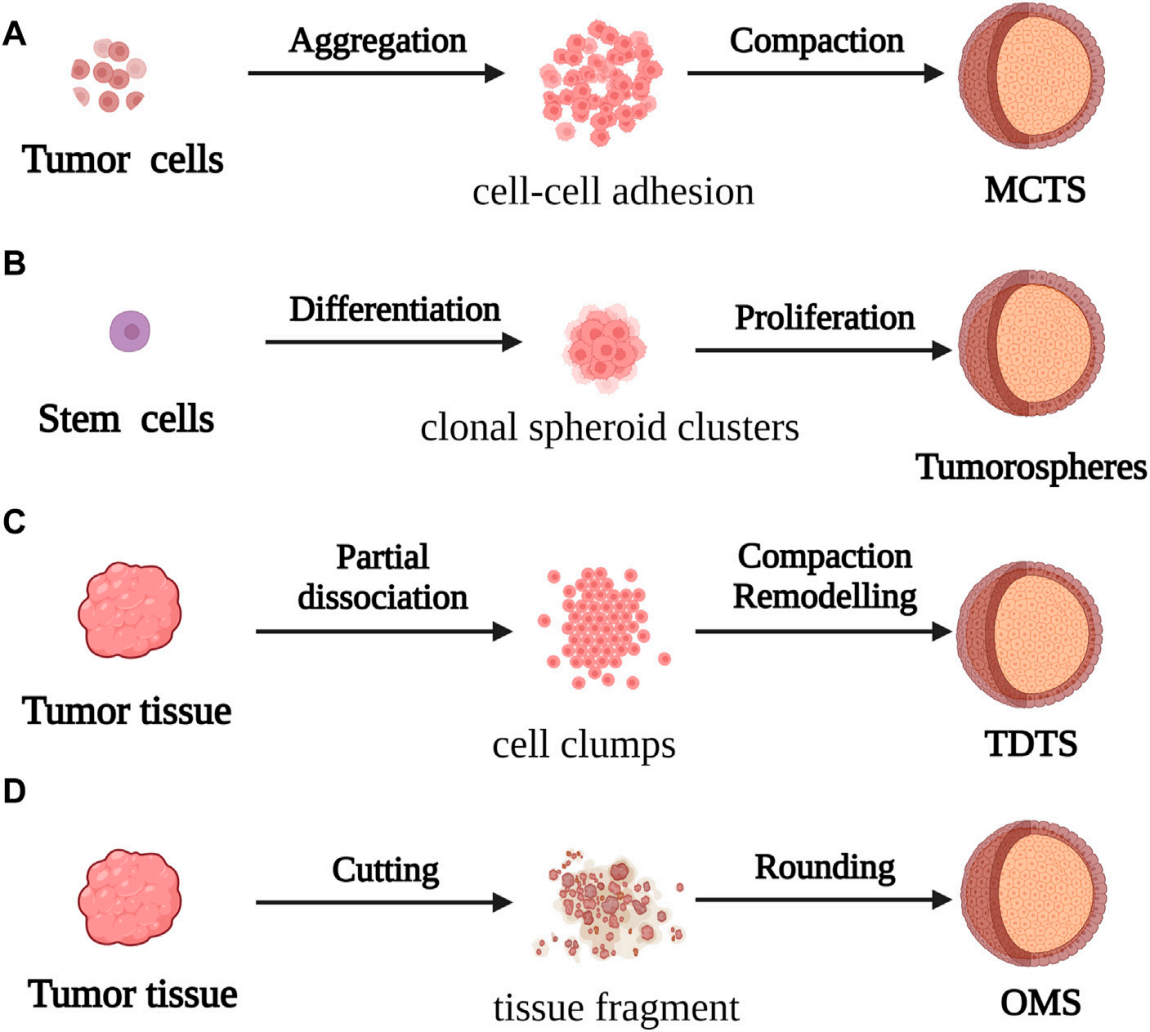
For the formation process of spherical cancer models **(A)** MCTS: Cell suspensions cultured under non-adherent conditions were aggregated and compacted to obtain MCTS; **(B)** Tumorospheres: Stem cells cultured under low-adherent conditions formed Tumorospheres by clonal proliferation **(C)** TDTS: Partial dissociation of tumor tissue and compaction/remodeling produced TDTS; **(D)** OMS: Cut tumor tissue aggregates formed OMS during culture under non-adherent conditions.

Spheroids are one of the most commonly used models in CRC research. They are constructed by suspending cancer cell lines or isolated tumor tissues from patients in CRC. They have a convenient mode of production and application, and are particularly effective for studying micrometastases or avascular tumors. Spheroid models can be categorized into four types according to the origin and morphology of the cancer cells from which they are derived. These categories include multicellular tumor spheroids (MCTS), tumorospheres, tissue-derived tumor spheres (TDTS), and organotypic multicellular spheroids (OMS; Figure 2) (Weiswald et al., 2015).

MCTS models, first constructed by Bauleth-Ramos, consist of colonic epithelia, human intestinal fiber cells, and human mononuclear cells, and are inoculated into hydrogel microwells to form the spheroid model (Inch et al., 1970; Bauleth-Ramos, T et al., 2020). MCTS models are similar to solid tumors in terms of the growth kinetics, metabolic rate, and resistance to chemotherapy and radiotherapy *in vivo* (Ivascu and Kubbies, 2006), and have been employed for screening and evaluating the efficacy of drugs. However, the variability of MCTS models makes it difficult to obtain repeatable and stable experimental data, which affects the use of these models in tumor research.

The tumorosphere model of CRC stem cells (CSCs) was used in the early 2000s for evaluating the differentiation capacity of tumors. However, because there are no morphological phenotypes associated with the phenotypic instability of CSCs, the tumorosphere model is unable to faithfully simulate the *in vivo* 3D framework and physiological condition of tumors (Valent et al., 2012).

The TDTS models consist of cancer and stromal cells, and are commonly used in studies on CRC. TDTS models of CRC tumors have a unique histological feature similar to the poorly differentiated globules produced by permanent cancer cell lines, and can fully simulate the characteristics of *in vitro* 3D cell culture models of CRC (Santini and Rainaldi, 1999; Weiswald et al., 2009).

OMS models are enriched in stem cells which can represent the complexity of parental tumor cells similar to *in vivo* tissues by forming an extracellular layer of epithelioid cells and an intracellular layer of mesenchymal cells, and thus maintaining the multicellular nature of CRC (Rajcevic et al., 2014). However, the difficulty of producing homogeneous spheres in a reproducible manner combined with the insufficiency of stable experimental data can prove to be a challenge during the application of the OMS model in CRC research and drug development.

#### 2.2.2 Organoids

**FIGURE 3:**
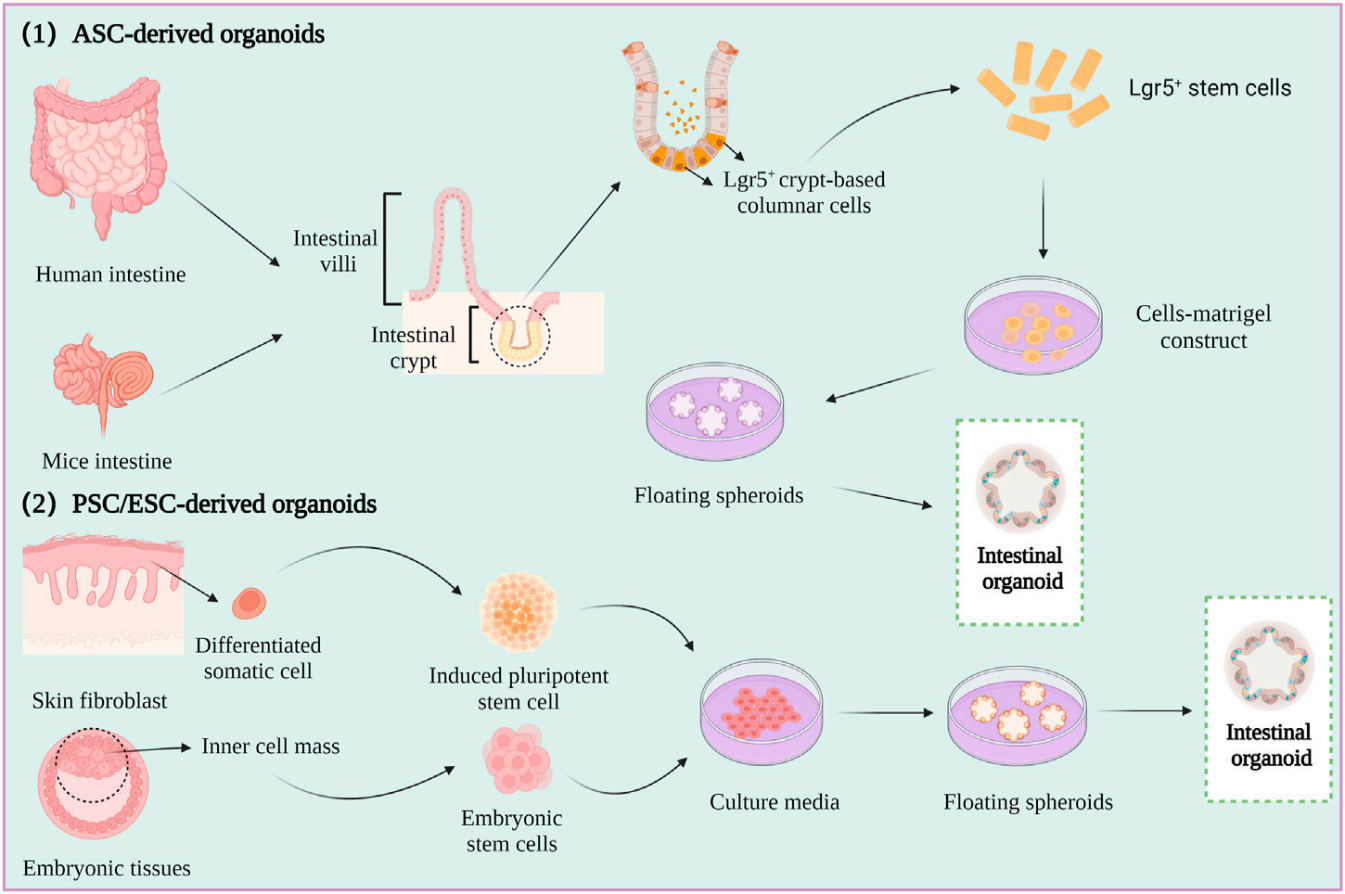
Intestine organoid cultures.

Spheroids are a simple experimental model that only partly represent the *in vivo* characteristics of tumor tissues*.* However, organoids are relatively complex three-dimensional (3D) culture models that are frequently used in CRC research. Organoids are self-organizing organotypic cultures that are produced from various stem cells, including tissue specific adult stem cells (ASCs), embryonic stem cells (ESCs), or induced pluripotent stem cells (iPSCs) (Fujii et al., 2018; Fujii and Sato, 2021). The stem cells are grown in matrigel 3D culture conditions to mimic the *in vivo* growth environment, and to produce stable, near-physiological epithelial structures (Figure 3) (Lancaster and knoblich, 2014; Huch and Koo, 2015).

The first intestinal epithelial 3D organoids were constructed by growing leucine-rich repeat-containing G-protein-coupled receptor 5 (LGR5^+^) intestinal stem cells in a medium containing stem cell niche restatement factors and tissue-specific growth factors (Sato et al., 2011). An increasing number of studies have described the formation of patient-derived organoids (PDOs) by culturing minced human CRC tumors in human intestinal stem cell medium (HISC), and the phenotype and genotype of the PDOs have been reported to be highly similar to those of the original tumor (Van et al., 2015; Vlachogiannis et al., 2018).

Organoids are typically used for investigating the mechanism underlying the development of CRC, screening anti-CRC drugs, and determining the efficacy and mechanism of action of drugs. However, there are various limitations to the application of organoids in studies on CRC, which are described hereafter. First, the current methods for organoid culture lack the technological means for maintaining the blood vessels, immune system, and peripheral nervous system of tumor cells, and organoids lacking these characteristics cannot be used in CRC research (Bredenoord et al., 2017). Second, as PDO models lack the cellular and acellular components of the TME of the original tumor, they cannot equivalently represent the *in vivo* environment of the tumor (Li X. et al., 2020). Third, there are no specific media for culturing organoids to date. Furthermore, it is unclear whether organoids can represent the overall heterogeneity of the tumor and all cell types in the tumor. Organoids can be applied to relevant studies by optimizing the culture conditions for maintaining the expression of genes related to microsatellite instability, B-Raf proto-oncogene, serine/threonine kinase (*BRAF*) mutations, poor differentiation, or mucinous phenotypes related to CRC. The application of organoids to CRC research can be improved by employing the co-culture model of organoids in which immune cells and mesenchymal cells are co-cultured for simulating the *in vivo* TME.

### 2.3 Application of cellular models of CRC

**FIGURE 4:**
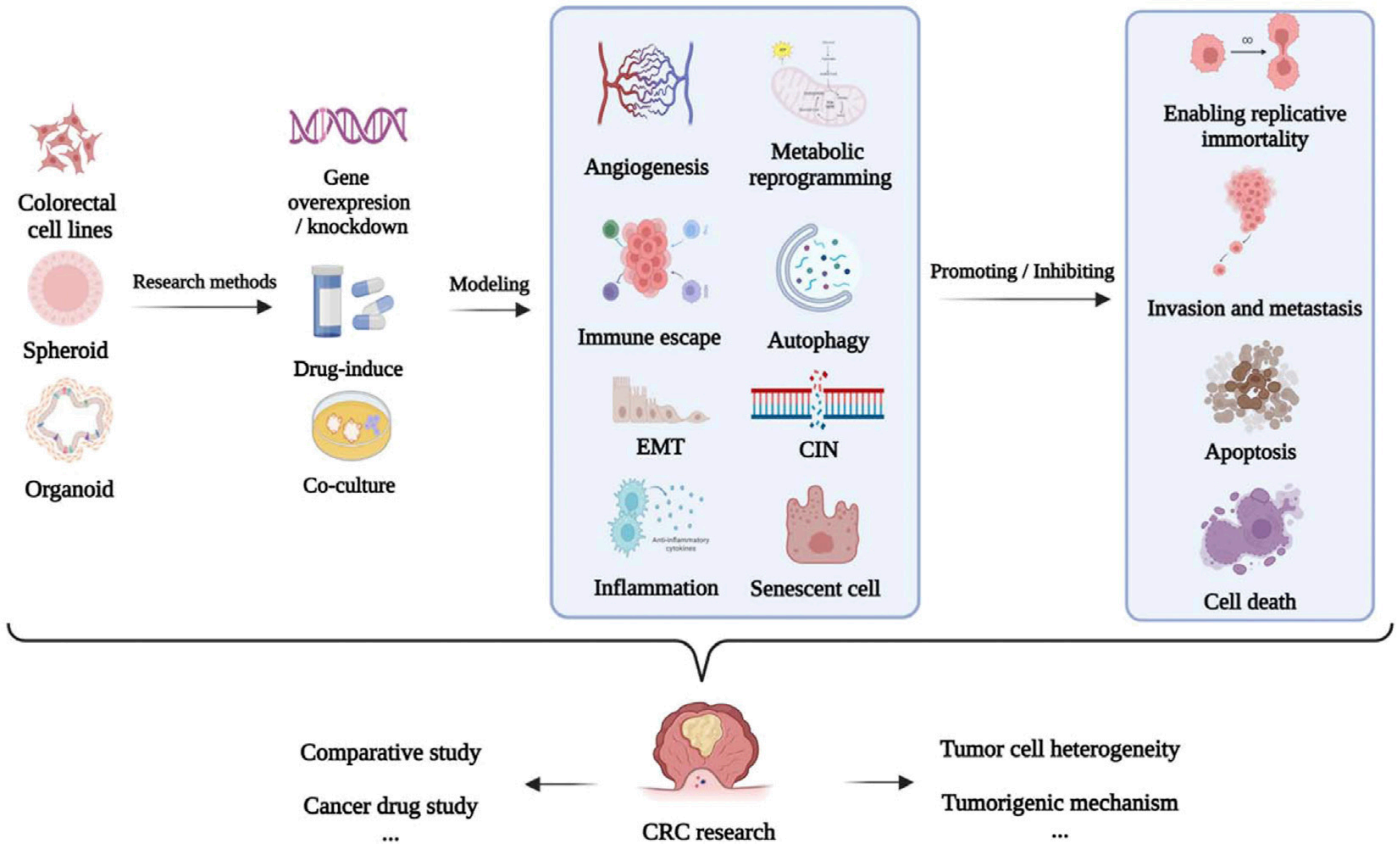
Application of CRC cellular models.

The establishment of models using the corresponding tumor cells is crucial for investigating the mechanism underlying the development of CRC and discovery of anti-CRC drugs (Senga and Grose, 2021). The applications of different cellular models of CRC according to the different molecular mechanisms underlying tumor formation, including epithelial–mesenchymal transition (EMT), apoptosis, invasion, metastasis, chromosome instability (CIN), and immune escape, are summarized in Table 1 and Figure 4.

**TABLE 1:** Applications of cellular models of CRC.

Mechanism being investigated	Research model	Cell lines	References
Apoptosis	Induction of apoptosis via the overexpression of neurofibromin (*NF2*), heterogeneous nuclear ribonucleoprotein L (*HNRNPL*), and other genes	HCT116 and SW620	Wu et al. (2020)
		HIEC, Caco2, HCT116, LoVo, and SW480	Zhao et al. (2021)
	Induction of apoptosis via the knockdown of ribosomal protein lateral stalk subunit P0 pseudogene 2 (*RPLP0P2*), Cadherin 17 (*CDH17*), and other genes	HCT116, HT29, SW480, and RKO	Yuan et al. (2021)
		KM12SM, KM12C, Colo320, HT29, RKO, and SW480	Tian et al. (2018)
	Inhibition of apoptosis via the knockdown of receptor interacting protein kinase 3 (*RIP3*)	SW480, HCT-116, RIP3^+/+−MEF^, and RIP3^−/−MEF^	Han et al. (2018)
	Inhibition of glycolysis and promotion of apoptosis via the knockdown of hypoxia-inducible factor-1α (*HIF-1α*)	FHC, CCD841 CoN, HT29, SW480, LoVo, HCT116, and SW620	Liu et al. (2019)
	Cu nanoparticles (CuNPs)-induced apoptosis of CRC cells	SW480	Ghasemi et al. (2020)
Autophagy	Inhibition of autophagy with chloroquine	HCT116 and SW480	Ma et al. (2020)
	Rapamycin-induced model of autophagy	KM12SM, KM12C, Colo320, HT29, RKO, and SW480	Tian et al. (2018)
Angiogenesis	Inhibition of angiogenesis via the knockdown of cellular-myelocytomatosis viral oncogene (*c-Myc*), vascular endothelial growth factor (*VEGF*), and other genes	HCT116	Yin et al. (2010)
	Co-culture of patient-derived cancer-associated fibroblasts (CAFs) and HUVECs	Patient-derived CAFs	Unterleuthner et al. (2020)
Invasion and metastasis	Promotion of invasion and metastasis via the overexpression of zinc-finger protein 326 (*ZNF326*), metastasis associated 1 family member 3 (*MTA3*), and other genes	SW480, SW620, CL187, and RKO	Yang et al. (2021)
		LoVo and HCT15	Jiao et al. (2017)
	Inhibition of invasion and metastasis via the overexpression of t-box transcription factor 5 (*TBX5*)	HT29, SW620, SW480, LoVo, and HCT116	Dong et al. (2020)
	Inhibition of invasive metastasis via the knockdown of sphingosine phosphate lyase 1 (*SGPL1*), forkhead Box O6 (*FOXO6*), and other genes	DLD-1, Caco-2, and CCD 841 CoN	Faqar et al. (2021)
		HCT116-CSC	Zou et al. (2022)
		NCM460, Caco2, HT29, HCT116, and SW480	Li et al. (2019)
	Co-culture of EMT-CRC cells and HUVECs	NCM460, LoVo, HCT-116, DLD-1, SW620, and SW480	Dou et al. (2021)
Metabolic reprogramming	Reprogramming of energy metabolism via the overexpression of mitochondrial citrate carrier solute carrier family 25 member 1 (*SLC25A1*), human kallikrein 2 (*HK2*), and other genes	NCM460, SW480, HCT116, SW620, LoVo, LS174T, and HT29	Yang et al. (2021a)
	Inhibition of metabolic reprogramming via *HIF-1α* knockout	HCT8, HCT15, HCT116, LoVo, SW480, SW1116, HT29, Caco-2, DLD-1, and T84	Dong et al. (2022)
Immune escape	Promotion of immune escape *via* lipopolysaccharide (LPS)-induced macrophage infiltration	HCT-8, HCT-116, SW620, SW480, DLD-1, CaCo-2, CT26, and HT-29	Liu et al. (2020a)
	Induction of immune escape via the overexpression of antigen-presenting-cell, B7 homolog x (*B7x*), and other genes	HCA-7, HT-29, 293T, and TALL-104	Cen et al. (2021)
		LoVo, Colo-205, SW480, SW620, HCT-116, CT-26, and MC-38	Li et al. (2020c)
Inflammation	LPS-induced model of inflammation	HCT116 and SW480	Zhu et al. (2019)
		—	Schafer and Werner (2008)
		Colon 26	Choo et al. (2005)
		—	Schottelius and Baldwin (1999)
	Induction of tumor necrosis factor-α (*TNF-*α), nuclear factor-kappa B (*NF-kB*), and other pro-inflammatory factors	Caco-2, HT29, SW480, SW48, and DLD1	Li et al. (2012)
		Volo	Tai et al. (2012)
EMT	Suppression of EMT via the knockdown of Pleckstrin homology-like domain family A member 2 (*PHLDA2*), SRY-Box transcription Factor 2 (*SOX2*), and other genes	HCT116 and SW480	Ma et al. (2020)
		SW480 and SW620	Zhu et al. (2021)
		HCT116 and LoVo	Qi et al. (2021)
		HCT116 and DLD-1	Ju et al. (2020)
		HCT116, SW480, HT29, and SW620	Hua et al. (2020)
	Induction of EMT via interleukin-6 (*IL-6*), *TNF-*α, and other inflammatory factors	SW480, SW620, and Caco-2	Rokavec et al. (2014)
		HCT116 and Caco-2	Wang et al. (2013)
	Induction of EMT via the overexpression of cryopyrin-associated periodic syndromes 1 (*CAPS1*), nuclear factor of activated T-cells (*NFATc1*), and other genes	FHC, HT29, SW480, SW620, and DLD1	Zhao et al. (2019)
		SW620, LoVo, Caco-2, SW480, HT29, HCT116, and DLD-1	Shen et al. (2021)
		HCT116	Li et al. (2021)
	Induction of EMT by X-ray irradiation	SW480	Lin et al. (2017)
Genomic instability/mutation (CIN)	—	CRC PDOs	Bolhaqueiro et al. (2019)
	Induction of CIN by DNA damage caused by the overexpression of iroquois homeobox gene 5 (*IRX5*), integrin-linked kinase (*ILK*), and other genes	SW480 and DLD-1	Sun et al. (2020)
		HCT116	Chadla et al. (2021)
Senescent cells	Induction of cellular senescence via the overexpression of lamin B1 (*LMNB1*), tribbles homolog 2 (*TRIB2*), and other genes	SW480, HT29, and IEC-6	Liu et al. (2013)
		HEK 293 T, SW48, and LoVo	Hou et al. (2018)
	Drug-induced senescence of CRC cells using oxaliplatin, adriamycin, aspirin, and other drugs	SW620 and HCT116	Jung et al. (2015)
		SW837, HCT116, and SW48	Tato-Costa et al. (2016)
		PROb and CT26	Seignez et al. (2014)
		HCT116	Vétillard et al. (2015)
		HCT116 and SW480	Zhang et al. (2011)
		C85	Dabrowska et al. (2011)
			Dabrowska et al. (2019)

## 3 CRC animal models based on experimental animals

The occurrence of diseases such as cancer that occur spontaneously in animals is largely attributed to genetic diversity and immune functions. Therefore, studying the methods for generating animal models of CRC can aid in elucidating the mechanisms underlying the development of cancer (Marian, 2004). Animal models can compensate for the limitations of cellular models that are incapable of simulating the mechanism underlying the development of CRC. Rat and murine models are the most frequently used animal models of CRC, and other animal models of CRC, including fruit fly, zebrafish, and pigs, are also commonly used as sentinels and preclinical models in CRC research.

### 3.1 Rodent models

Rodent models are conducive tools for conducting cancer research, and are extensively used for elucidating the etiopathogenesis and molecular mechanisms underlying the development of CRC. Previous studies have demonstrated that the protein-coding genes of mice and humans share high homogeneity (Mouse Genome Sequencing Consortium, 2002). Additionally, the use of murine models is advantageous owing to the fact that mice have a short intergenerational interval, high reproducibility, and similar genetic background and formula as humans, compared to other animal models. Murine models of CRC can therefore be used as effective tools for studying the mechanism underlying the pathogenesis of CRC and determining novel strategies for the prevention and treatment of CRC (Doyle et al., 2012).

Transgenic mice models can serve as effective tools for preclinical evaluation and screening during the optimization and development of anticancer drugs. Mutations in *APC* (adenomatous polyposis coli) are commonly inherited in adenoma-carcinoma transitions observed during the development of CRC (Van et al., 2000). Additionally, the absence of mutations in DNA mismatch repair (MMR) genes increases deletion mutations in *APC*, which accelerates the formation of adenomas (Huang et al., 2004). It has been reported that mutations in tumor protein 53 (*p53*), Kirsten rats arcomaviral oncogene homolog (*KRAS*), phosphatidylinositol-4,5-bisphosphate 3-kinase catalytic subunit alpha (*PIK3CA*), F-box and WD repeat domain containing 7 (*FBXW7*), SMAD family member 4 (*SMAD4*), transcription factor 7-like 2 (*TCF7L2*), NRAS proto-oncogene (*NRAS*), AT-rich interaction domain 1 A (*ARID1A*), SRY-box transcription factor 9 (*SOX9*), and APC membrane recruitment protein 1 (*FAM123B*) can also increase the risk of CRC (Cancer Genome Atlas Network, 2012). Transgenic murine models are extensively used for studying the occurrence and elimination of tumors, underlying molecular pathways, and genomic regulation via gain-of-function or loss-of-function mutations in oncogenes and cancer suppressor genes.

**FIGURE 5:**
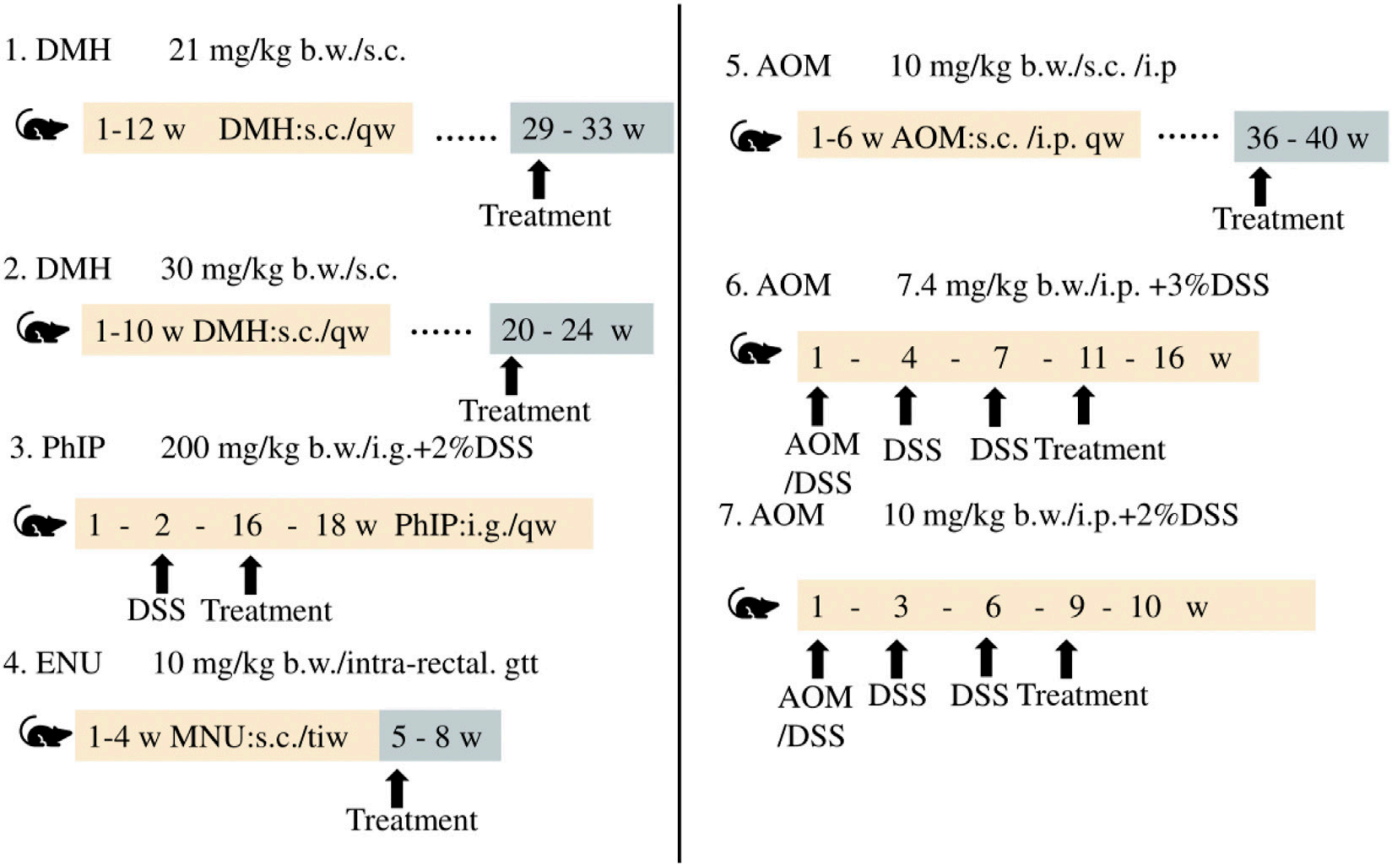
Chemically induced CRC animal models.

CRC is caused by various risk factors, including poor dietary habits, environment, exposure to carcinogenic chemicals, and other factors (Hecht, 2003; Mehta et al., 2017). Animal models of CRC generated by treatment with chemicals serve as effective models in studies aimed at determining novel therapeutic approaches and investigating the diagnosis, prognosis, and identification of predictive markers. The differences among the methods and duration of treatment for inducing CRC with different chemical agents are depicted in Figure 5.

The use of chemical agents for generating models of CRC requires a long duration and these models have longer experimental cycles. Mofikawa et al. established the first orthotopic transplantation model of CRC in 1986 by transplanting human CRC cells under the cecal wall of nude mice. This shortened the period of study using animal models of CRC, and initiated the establishment of tumor transplantation models. Table 2 summarizes the different murine models of CRC, and describes their scope of application and limitations in tumor research.

**TABLE 2:** Murine models of CRC.

Model	Strategy for model generation	Pathological mechanism	Detailed methodology	Range of application	Limitations	References
Spontaneous animal model of CRC	Mutant animal models of CRC	Proliferation	Mutation in *APC*	FAP model for studying hereditary CRC	Survival time < 4 months, tumor formation in small intestine, difficulty in metastasis	Moser et al. (1990)
						Shoemaker et al. (1997)
						Shoemaker et al. (1998)
						Barker et al. (2007)
			Mutation in *APC/Cre*	Induction of colorectal adenoma	Difficulty in metastasis	Robanus-Maandag et al. (2010)
						Chen et al. (2020)
			Mutations in *Mlh1, Msh2, Msh3, Msh6,* and *Pms2*	Hereditary nonpolyposis CRC (HNPCC)	Multi-tissue tumors, difficulty in metastasis	Lynch et al. (1997)
						Papadopoulos and Lindblom (1997)
						Manceau et al. (2011)
			Mutation in *SMAD4*	Familial juvenile polyposis model, acceleration of tumor development	Difficulty in metastasis	Takaku et al. (1998)
						Lu et al. (1998)
			Mutation in *KRAS*	Induction of colonic hyperplasia and generation of aberrant crypt foci (ACF) carcinogenesis model	CRC cannot be induced by mutations in single genes, but is induced in combination with other gene mutations that induce carcinogenesis and enhance the incidence of CRC.	Bos et al. (1987)
						Campbell et al. (1998)
						Jen et al. (1994)
						Janssen et al. (2002)
						Janssen et al. (2006)
						Calcagno et al. (2008)
			Mutation in *PIK3CA*	Induction of colon adenoma	Single mutations generally do not induce CRC.	Juric et al. (2018)
		Invasion and metastasis	Mutation in *FBXW7*	Model of highly invasive colorectal cancer	Single mutations generally do not induce CRC.	Mao et al. (2004)
			Mutation in *p53*	Induction of distal intestinal tumor	Single mutations generally do not induce CRC.	Nakayama et al. (2017)
						Kadosh et al. (2020)
Diet- and chemical-induced models of CRC	Diet-induced models of CRC	Inflammation	High-fat diet (HFD)/western diet (NMD)	Colorectal barrier dysfunction and inflammation, invasive adenocarcinoma	Requires a long duration and has a low carcinogenic efficiency	Itano et al. (2012)
						Yu et al. (2022)
	Chemical-induced models of CRC		2,4,6-Trinitro-benzenesulfonic acid (TNBS)	Induction of colitis-driven CRC	Cannot be used alone, necessary to break the intestinal mucosal screen before use, mortality rate of modeling is high	Scheiffele and Fuss (2002)
			Anaerobic oxidation of methane (AOM) + dextran sodium sulfate (DSS)	Tumors driven by colitis, induced distal CRC	The modeling rate is low and molding time is uncertain	Neufert et al. (2007)
						De-Robertis et al. (2011)
						Liang et al. (2017)
						Sun et al. (2022)
		Proliferation	AOM	ACF and CRC epithelial tumor model	The period of modeling is long and time-consuming, cannot be used for studying CRC metastases	Femia and Caderni (2008)
						Izzo et al. (2008)
						Orlando et al. (2008)
			1,2 Dimethyl hydrazine (DMH)	Human sporadic CRC research model, tumorigenicity specificity	Requires a long time and has a low carcinogenic efficiency	Ma et al. (1996)
						Kissow et al. (2012)
						Aranganathan and Nalini (2013)
			Parahydrogen-induced polarization (PhIP)	ACF-induced rat model	Low incidence, long study cycle	Ito et al. (1991)
						Tanaka et al. (2005)
			3,2′-Dimethyl-4-Aminobiphenyl (DMAB)	Induced colon and small intestinal carcinogenesis	Requires multiple administration, low specificity	Reddy and Mori (1981)
						Reddy (1998)
		CIN	N-ethyl-N-nitrosourea (ENU)/N- methyl -N-nitrosourea (MNU)/N-methyl-N-nitrosoguanidine (MNNG)	Induced distal CRC model	Induced mutations are random and drug volume quantification is difficult	Huang et al. (2020)
Animal model of transplanted CRC	Animal model of orthotopic tumor transplantation	Invasion and metastasis	Cecal transplantation	Induction of primary CRC that can metastasize to local lymphatic vessels, lungs, and liver	Risk of laparotomy is high in this model. CRC originates from the mucosa, and whether tumor metastasis results from the overflow of intraperitoneal cells cannot be excluded	Talmadge et al. (2007)
						Martin et al. (2013)
						Lee et al. (2014)
						O'Rourke et al. (2017)
	Animal model of ectopic tumor transplantation		Spleen planting	Study of advanced CRC	The operation is complex and requires highly advanced technical skills	Kasuya et al. (2005)
						Bai et al. (2015)
						Yang et al. (2021c)
			Tail vein injection	Lung metastasis model of CRC	Differs from human CRC metastasis, multiple metastases are prone to occur	Wang et al. (2020)
			Liver implantation	Liver metastasis model of CRC	Only the late metastatic process of CRC is simulated; tumor forms only at the site of implantation	Panis and Nordlinger (1991)
						Kopetz et al. (2009)
						Roque et al. (2019)
			Intraperitoneal injection of CRC cell for inducing metastasis	Peritoneal metastasis model of CRC	Unsuitable for studying early metastasis of lymph nodes in CRC.	Li et al. (2016)
		Proliferation	Hypodermic implantation	Real-time monitoring of CRC growth	Cannot simulate the *in situ* growth of CRC, not easy to study tumor invasion and metastasis	Rygaard and poulsen (1969)
						Lehmann et al. (2017)

### 3.2 Other animal models of CRC

In addition to rodents, invertebrates such as fruit fly can be used for personalized diagnosis and developing potential therapeutic strategies for CRC. Vertebrates such as zebrafish, dogs, cats, pigs, and non-human primates are also used in studies on CRC. The advantages and disadvantages of the different animal models used in CRC research are summarized in Table 3.

**TABLE 3:** Other animal models of CRC.

Classification	Animal	Advantages	Disadvantages	References
Invertebrate	*Drosophila melanogaster* (fruit fly)	The model can represent the composition of mammalian intestinal cells, aids in avoiding cancer heterogeneity	The model has no acquired immune function and has a short life cycle. It is impossible to simulate the complexity of tumor development	Bhandari and Shashidhara, 2001 Martorell et al., 2014
Vertebrate	*Danio rerio* (zebrafish)	Histopathological features of intestinal tumors are similar to those of human tumors. High transparency of seedlings, small size, short developmental cycle, *in vitro* fertilization, and large number of eggs. Requires small experimental dosage and is less time-consuming	The culture temperature is inconsistent with the growth temperature of tumor cells. Long-term tumor transplantation experiments cannot be performed	Amatruda et al. (2002)
				Trede et al. (2004)
				Haldi et al. (2006)
				Brugman et al. (2009)
				Paquette et al. (2013)
	*Canis lupus familiaris* (Dog)	The model has a similar physiological structure to humans, and the mechanism of pathogenesis is similar to sporadic CRC in humans. Gentle character, good experimental coordination, and repeatability	Long duration of modeling, observational inconveniences, not suitable for acute experiments	Kamano et al. (1981)
				Kamano et al. (1983)
				Youmans et al. (2012)
	*Felis catus* (Domestic cat)	The histological subtype of the model is similar to that of advanced CRC in humans. Model can be used for studying the germination of intestinal tumor in CRC.	Low incidence, tumors mostly occur in the small intestine	Uneyama et al. (2021)
				Groll et al. (2021)
	*Sus scrofa* (Pig)	The anatomical structure of the small intestine is similar to that of humans. Model has a moderate size and long life. The progression and accumulation of mutations in CRC can be monitored by colonoscopy screening	The model cannot be used to study acute CRC as the process of cancer formation is slow	Llanos et al. (2006)
				Sangild et al. (2006)
				Flisikowska et al. (2012)
				Dean (2013)
				Flisikowska et al. (2017)
				Gonzalez et al. (2019)
	*Ovis aries* (Sheep)	Cellular differentiation in the model is similar to that of colon adenocarcinoma in humans. Model can be used to study advanced CRC.	Adenocarcinoma develops in the small intestine	Munday et al. (2006)
	*Macaca mulatta* (Rhesus monkey)	Shares high genomic homology with humans; anatomical and physiological similarities. Shares same clinicopathological features as human Lynch syndrome	Research cycle or modeling time-consuming	Bakken et al. (2016)
				Dray et al. (2018)
				Ozirmak et al. (2022)

### 3.3 Application of animal models of CRC

The carcinogenesis of CRC is affected by several contributing factors. The selection of the animal model of CRC depends on the purpose of the study, as summarized in Table 4.

**TABLE 4:** Applications of animal models of CRC.

Purpose of study	Research methods/models	References
Studying apoptosis in CRC	Investigation of apoptosis in CRC with CRC xenograft models	Han et al. (2018)
		Li et al. (2020a)
Investigation of angiogenesis in CRC	Studying the effect of AOM/DSS-induced expression of severe acute respiratory infection (*SARI*) gene on angiogenesis in CRC	Dai et al. (2016)
	DMH/DSS-induced expression of CRC angiogenesis factor in rat model	Liu et al. (2015a)
	Induction of tumor angiogenesis *in vivo via* the expression of *VEGF* and interleukin-8 (*IL-8*)	Liu et al. (2015b)
	Studying angiogenesis in CRC xenografts following induction with drugs, C-X-C motif chemokine ligand 12 (*CXCL12*), and *CXCL11*	Rupertus et al. (2014)
		Yu et al. (2005)
		Jakopovic et al. (2020)
	Drug-induced *in vivo* inhibition of angiogenesis	Petrović et al. (2020)
	Dickkopf associated protein 2 (*DKK2*)-induced angiogenesis in CRC xenografts	Ding et al. (2016)
		Deng et al. (2019)
	Inhibition of angiogenesis by potentially inappropriate medication (PIM) kinase in orthotopically transplanted CRC tumors	Casillas et al. (2018)
	Induction of angiogenesis by hepatectomy in CRC xenografts	Lo et al. (2018)
	*EG-VEGF* induced angiogenesis in orthotopically transplanted CRC tumors	Goi et al. (2004)
Investigation of metabolic reprogramming in CRC	Induction of metabolic reprogramming in CRC xenograft model using hexokinase, free fatty acid (FFA), acetyl coenzyme A, citrate, and other agents	Bu et al. (2018)
		Wang et al. (2018)
		Dong et al. (2022)
		Zhang et al. (2022)
	AOM/DSS-induced CRC model of metabolic reprogramming	Wu et al. (2020)
		Yin et al. (2021)
	Initiation of metabolic reprogramming by DSS-induced inflammation	Qu et al. (2017)
Study of invasion and metastasis in CRC	CRC xenograft model for studying invasion and metastasis in CRC	Rokavec et al. (2014)
		Erreni et al. (2016)
		Li et al. (2019b)
Study of immune escape in CRC	Gene mutation-induced model of immune escape	Xing et al. (2021)
		Wei et al. (2022)
	Generation of immune escape model by ablation of zebrafish macrophages using chlorophosphonate liposomes	Póvoa et al. (2021)
Study of inflammation in CRC	TNBS/oxazolone/DSS-induced inflammatory CRC	Wirtz et al. (2007)
	LPS/DSS-induced inflammation	Garlanda et al. (2004)
	DSS-induced inflammation of intestinal epithelium and mucosa	Mashimo et al. (1996)
		Van et al. (2006)
	DSS/AOM-induced inflammation in sporadic CRC	De et al. (2019)
		Liang et al. (2017)
	TNBS-induced inflammation	Scheiffele and Fuss (2002)
	DMH-induced inflammation	Kumar et al. (2019)
	Radiofrequency ablation (RFA)-induced inflammation	Shi et al. (2019)
	HFD-induced inflammation	Hu et al. (2021)
	Gene mutation-induced inflammatory CRC	Puppa et al. (2011)
		De et al. (2020)
	High-iron diet-induced inflammatory CRC	Seril et al. (2006)
Investigation of the mechanism of EMT in CRC	Induction of EMT models via mutations/overexpression/knockdown *p* rostate transmembrane protein androgen induced 1 (*PMEPA1*), SOX2, histone deacetylase 1 (*HDAC1*), and other genes	Wang et al. (2014)
		Matsuda et al. (2016)
		Li et al. (2017a)
		Zhuang et al. (2018)
		Yang et al. (2019)
		Zhang et al. (2019)
		Liu et al. (2020b)
		Shen et al. (2021)
		Qi et al. (2021)
		Zhu et al. (2021)
		Liu et al. (2020)
	Transforming growth factor-β (*TGF-β*)-induced model of EMT	Li et al. (2021)
	Tumor EMT-induced metastatic model of CRC	Adams et al., 2021
Epigenetic reprogramming	CRC xenograft model for studying epigenetic reprogramming in CRC	Kodach et al. (2021)
	Induction of gene mutation for studying epigenetic reprogramming in CRC	Hashimoto et al. (2017)
Study of cell aging in CRC	Xenotransplantation model for studying cellular aging in CRC	Gao et al. (2010)
		Liu et al. (2013)
		Mikuła et al. (2015)
		Hou et al. (2018)
	DMH/DSS-induced model of cellular aging	Liu et al. (2013)
	AOM/DSS-induced model of cellular aging	Foersch et al. (2015)
Polymorphic microbiota	AOM/DSS-induced model for studying composition of intestinal microbiota	Wu et al. (2016)

Traditional Chinese medicine (TCM) and western medicine are two different medical theoretical systems. The research model based on the etiological mechanism theory of TCM is applied to animal studies with TCM syndrome, as shown in Table 5.

**TABLE 5:** Applications of animal models of CRC.

TCM syndrome	Research methods/models	References
CRC with spleen qi deficiency syndromeHou et al., 2018 (SDS)	Restricted feeding/fatigue/purging + hypodermic implantation of C26 tumor cells to establish a spleen deficiency with cachexia model	Zhang et al. (2020)
CRC with damp-heat syndrome (DHS)	HFD/AOM/DSS-induced malignant tumor (stasis-toxin) model	Cao and Zhou (2020)
		Huang et al. (2022)
CRC with internal retention of toxin stagnation syndrome (IRTSS)	LPS tail vein and peritoneal injection + hypodermic implantation of C26 tumor cells to establish colorectal tumor-bearing with syndrome of heat-toxicity and blood stasis model	Li et al. (2017b)

## 4 Conclusions and future directions

Understanding the inherent advantages and limitations of the different models of CRC, and the appropriate application of these models in drug development and studies on the mechanism of tumor occurrence and development are important in CRC research.

Human cell lines and xenograft models have been extensively employed over the past few decades owing to their low cost and ease of application. However, these models are incapable of reproducing the heterogeneity of CRC tumors (Harma et al., 2010). The cell co-culture technique can overcome the limitations of monolayer cell culture, and enables the construction of *in vitro* physiological or pathological models that closely represent the *in vivo* condition, and can be used for studying the interactions between cells, and between cells and the culture environment. It has been reported that 3D models can mimic the physiological characteristics of parental tumors, including tumor heterogeneity (Li et al., 2019). However, the shape, size, and activity of organoids are different under the same culture conditions, and the matrix limits the penetration of drugs and hinders drug screening (Zhao et al., 2020). It is therefore imperative to construct a model that closely represents the characteristics of CRC *in vivo*.

The intestinal microarray platforms used in CRC research, which consist of intestinal organoids and organic chips, can summarize the important structural features and functions of the natural duodenum. This platform can be applied for studying drug conveyance, metabolism, and drug-drug interactions (Kasendra et al., 2018). Multi-locus transfer chips consist of multiple 3D organoids that connect the CRC-like organs, liver, lungs, and endothelial flow via recirculating fluid systems, and enables cell tracking by fluorescence imaging technology. The transfer sites of CRC cells are also included in multi-locus transfer chips (Aleman and Skardal, 2019).

Animal models of CRC have been widely used for studying the complexity of CRC. There are primarily two types of animal models, namely, *in situ* models and the cell and tissue transplantation models of CRC. Owing to the relatively simple modeling approach of human tumor xenotransplantation, this model is presently widely used for studying the efficacy of anti-CRC drugs. The effects of CRC xenotransplantation can be closely related to clinical activity via the rational application of these models. For instance, genetically engineered murine models have been used for studying the progression of tissue-specific molecular changes in CRC by determining the effect of specific molecular targets. Chemical induced-CRC animal model is one of the most commonly CRC models, in which CAC model is usually induced by AOM/DSS to study the mechanism of inflammation related-tumorigenesis and development (Zeng et al., 2022). The CRC model with TCM syndrome is an artificial disease and syndrome experimental animal model created by simulating and replicating characteristics of human disease prototype according to TCM theory. An animal model combining with CRC and TCM syndromes might be useful to mimic the clinical characteristics of CRC patients with TCM syndrome (Zhang et al., 2020). Mouse is the commonly used to the models mentioned above, however, it is increasingly accepted that the use of larger animal models, especially dogs and pigs, can provide deeper insights in cancer research (Croker et al., 2009).

The application of molecular tools and genetic strategies has aided the advancement of cancer research, and the cellular and animal models of CRC are being continually improved. Further understanding of the genetic and epigenetic events in CRC, including the alterations in molecular networks associated with the initial stages of development, are facilitated by high-resolution approaches.

Although CRC research has advanced immensely in recent years, several clinical issues remain to be resolved to date, which is partly attributed to the absence of suitable preclinical research models. The application of *in vivo* and *in vitro* models in CRC research, combined with advanced scientific techniques for simulating a more realistic tumor environment *in vivo* and *in vitro*, can help replicate the complex scenarios of tumor occurrence and development, identify novel therapeutic approaches for inhibiting tumor growth, and elucidate the molecular mechanisms underlying tumor formation.

## Figures and Tables

**Table:** 

**ACF**	Aberrant crypt foci
**AOM**	Anaerobic oxidation of methane
**APC**	Adenomatous polyposis coli
**ARID1A**	AT-rich interaction domain 1A
**ASCs**	Adult stem cells
**B7x**	B7 homolog x
**BRAF**	B-Raf proto-oncogene, serine/threonine kinase
**CAFs**	Cancer-associated fibroblasts
**CAPS1**	Cryopyrin-associated periodic syndromes 1
**CDH17**	Cadherin 17
**CIN**	Chromosome instability
**C-Myc**	Cellular-myelocytomatosis viral oncogene
**CRC**	Colorectal cancer
**CSCs**	Colorectal cancer stem cells
**CXCL12**	C-X-C motif chemokine ligand 12
**DHS**	Damp-heat syndrome
**DKK2**	Dickkopf associated protein 2
**DMH**	1,2 Dimethyl hydrazine
**DMAB**	3,2′-Dimethyl-4-Aminobiphenyl
**DSS**	Dextran sodium sulfate
**EMT**	Epithelial-mesenchymal transition
**ENU**	N-ethyl-N-nitrosourea
**ESCs**	Embryonic stem cells
**FAM123B**	APC membrane recruitment protein 1
**FBXW7**	F-box and WD repeat domain containing 7
**FFA**	Free fatty acids
**FOXO6**	Forkhead Box O6
**HDAC1**	Histone deacetylase 1
**HFD**	High-fat diet
**HIF-1α**	Hypoxia-inducible factor-1α
**HISC**	Human intestinal stem cell
**HK2**	Human kallikrein 2
**HNRNPL**	Heterogeneous nuclear ribonucleoprotein L
**HNPCC**	Hereditary nonpolyposis colorectal cancer
**ILK**	Integrin-linked kinase
**iPSCs**	Induced pluripotent stem cells
**IL-6**	Interleukin-6
**IL-8**	Interleukin-8
**IRTSS**	Internal retention of toxin stagnation syndrome
**IRX5**	Iroquois homeobox gene 5
**KRAS**	Kirsten rats arcomaviral oncogene homolog
**LGR5^+^**	Leucine-rich repeat-containing G-protein-coupled receptor 5
**LMNB1**	Lamin B1
**LPS**	Lipopolysaccharide
**MCTS**	Multicellular tumor spheroids
**MNU**	N-methyl-N-nitrosourea
**MNNG**	N-methyl-N-nitrosoguanidine
**MTA3**	Metastasis associated 1 family member 3
**NFATc1**	Nuclear factor of activated T-cells
**NF2**	Neurofibromin 2
**NF-kB**	Nuclear factor-kappa B
**NRAS**	NRAS proto-oncogene, GTPase
**OMS**	Organotypic multicellular spheroids
**p53**	Tumor protein 53
**PDOs**	Patient-derived organoids
**PhIP**	Parahydrogen-induced polarization
**PHLDA2**	Pleckstrin homology-like domain family A member 2
**PIM**	Potentially inappropriate medication
**PIK3CA**	Phosphatidylinositol-4,5-bisphosphate 3-kinase catalytic subunit alpha
**PMEPA1**	Prostate transmembrane protein androgen induced 1
**RIP3**	Receptor interacting protein kinase 3
**RPLP0P2**	Ribosomal protein lateral stalk subunit P0 pseudogene 2
**SARI**	Severe acute respiratory infection
**SDS**	Spleen qi deficiency syndrome
**SGPL1**	Sphingosine phosphate lyase 1
**SLC25A1**	Solute carrier family 25 member 1
**SMAD4**	SMAD family member 4
**SOX2**	SRY-box transcription Factor 2
**SOX9**	SRY-box transcription factor 9
**TBX5**	T-box transcription factor 5
**TCF7L2**	Transcription factor 7-like 2
**TCM**	Traditional Chinese medicine
**TDTS**	Tissue-derived tumor spheres
**TGF-β**	Transforming growth factor β
**TME**	Tumor microenvironment
**TNBS**	2,4,6-Trinitro-benzenesulfonic acid
**TNF-α**	Tumor necrosis factor-α
**TRIB2**	Tribbles homolog 2
**VEGF**	Vascular endothelial growth factor
**ZNF326**	Zinc-finger protein 326
